# Pesticide Residues and Risk Assessment from Monitoring Programs in the Largest Production Area of Leafy Vegetables in South Korea: A 15-Year Study

**DOI:** 10.3390/foods10020425

**Published:** 2021-02-15

**Authors:** Duck Woong Park, Yong Shik Yang, Yeong-Un Lee, Sue Ji Han, Hye Jin Kim, Sun-Hee Kim, Jong Pil Kim, Sun Ju Cho, Davin Lee, Nanju Song, Yujin Han, Hyo Hee Kim, Bae-Sik Cho, Jae Keun Chung, Ae Gyeong Kim

**Affiliations:** 1 Gakhwa Agricultural Products Inspection Center, Health and Environment Research Institute of Gwangju, 260, Dongmun-daero, Buk-gu, Gwangju 61138, Korea; youmeandus@naver.com (Y.S.Y.); gloryyw@mail.go.kr (Y.-U.L.); hjshj94@mail.go.kr (S.J.H.); jini2020@mail.go.kr (H.J.K.); aegyung@korea.kr (A.G.K.); 2 Seobu Agro-Fishery Products Inspection Center, Health and Environment Research Institute of Gwangju, 16, Maewol 2-ro, Seo-gu, Gwangju 62072, Korea; sunny1989@korea.kr (S.-H.K.); kjp8498@korea.kr (J.P.K.); sj0426@korea.kr (S.J.C.); dabin64@korea.kr (D.L.); snj0137@korea.kr (N.S.); hyj100@korea.kr (Y.H.); hhee1664@korea.kr (H.H.K.); 3 Health and Environment Research Institute of Gwangju, 584, Mujin-daero, Seo-gu, Gwangju 61954, Korea; foodcbs@korea.kr (B.-S.C.); jkchung@korea.kr (J.K.C.)

**Keywords:** pesticide residue, leafy vegetable, South Korea, azoxystrobin, procymidone, risk assessment

## Abstract

Leafy vegetables are widely consumed in South Korea, especially in the form of kimchi and namul (seasoned vegetables) and are used for wrapping meat. Therefore, the management of pesticide residues in leafy vegetables is very important. A total of 17,977 samples (49 leafy vegetables) were mainly collected in the largest production area of leafy vegetables (Gwangju Metropolitan City and Chonnam Province) in South Korea. They were analyzed within the government’s monitoring programs (Gwangju Metropolitan City) of pesticide residues between 2005 and 2019. Pesticide residues were found in 2815 samples (15.7%), and 426 samples (2.4%) from among these exceeded the specified maximum residue limits (MRLs). Samples exceeding the MRLs were mostly detected in spinach, ssamchoo (brassica lee ssp. namai), crown daisy, lettuce, and perilla leaves. Azoxystrobin, dimethomorph, and procymidone were the most frequently detected pesticides. However, procymidone, diniconazole, and lufenuron were found to most frequently exceed the MRLs. The rate of MRLs exceeding has been managed below the average (2.4%) more recently than in the past in this area. Further, leafy vegetables with the most violations of the MRLs in our study in South Korea were not harmful to health by a risk assessment (the range of the hazard index was 0.001–7.6%).

## 1. Introduction

A pesticide is any substance or a mixture of chemical substances and is used to protect crops against insects, other pests, fungi, and weeds [1,2]. Further, pesticides are used to modify a plant growth regulator used as a defoliant or desiccant [3]. There are many different kinds of pesticides. Each pesticide is meant to be effective on specific pests. The “-cide” suffix is derived from the Latin word caedere, meaning to kill [4]. The term pesticide includes all of the following: fungicides, molluscicides, nematicides, insecticides, herbicides, piscicides, avicides, rodenticides, bactericides, insect repellents, animal repellents, and antimicrobials [5]. Herbicides are the most common type of pesticide used, accounting for almost 80% of all pesticides used by the agriculture sector [6]. Pesticides may remain on or in food after spraying crops. These are known as “pesticide residues” [7]. Many problems arise by eating food (from meat, poultry, and fish to vegetable oils, nuts, and various fruits and vegetables, etc.) containing pesticide residues. Health effects of pesticide residues may be acute or chronic in those who are exposed depending on the quantity and ways. They may induce negative health effects that have been associated with the immune or nervous system, reproduction, and cancer because pesticides are potentially toxic [8]. Exposure to pesticide residues through food intake can harmfully influence the central nervous system because many pesticides can kill pests by disrupting the nervous system. Organophosphates used mostly in the category of insecticides are especially toxic to the nervous system (inhibitory effects on cholinesterase enzymes) [9]. 

Despite concerns about their use, pesticides have many advantages, such as the prevention of, reduction in, and elimination of pests at different steps of cultivation and post-harvest in the process of agricultural production. Pesticide usage can help enhance the yield and quality of the produce [10]. Therefore, countries worldwide have been making continuous efforts to ensure proper and safe use of pesticides for many years. In South Korea, the pesticide residue monitoring program began in 1968, and the maximum residue limits (MRLs) regulations for 17 pesticides were first established in South Korea in 1988 [11]. South Korean MRLs were established based on supervised pesticide residue trials. Ensuring safety against residual pesticides in vegetables is especially important in South Korea because vegetables are a very important part of the South Korean diet [12]. Basic Korean dishes such as kimchi (traditionally fermented Korean food) and namul (seasoned vegetables) use plenty of vegetables, especially raw leafy vegetables. These are used to wrap grilled or boiled meat [13]. Pesticides are highly likely to remain in leafy vegetables because leafy vegetables have broad surface areas [14]. 

Globally, leafy vegetables (raw, boiled, or steamed) consumed account for 2% of total vegetables. Further, these are thought to have a relatively greater health impact than cereal ingestion [14]. Therefore, the management of residual pesticides in the case of leafy vegetables is more important than other types of vegetables. Some leafy vegetables, such as spinach, napa cabbage, and lettuce, are also exported at a mean of roughly 10 million tons/year in South Korea (ATKATI, 2015) [15]. 

The yields of Gwangju and Chonnam Province account for the largest proportion (38.6%) (Chonnam area accounts for 30.4%) of all types of leafy vegetables produced in South Korea over the past 3 years (KOSTAT 2019) [16]. Therefore, it is necessary to have surveillance programs to analyze the trend of pesticide residues in this area of leafy vegetables. Some studies were conducted on pesticide residues of vegetables for a period of not more than five years in South Korea [13,17,18,19,20,21]. These papers mainly dealt with research on residual pesticides for the entire agricultural product range and health risk assessment. However, this is the first long-term study providing results covering more than a decade from South Korea’s largest leafy vegetable-producing area. Moreover, recently, during a residual pesticide test of leafy vegetables, we found several changes in detection compared to the past. Therefore, we thought it necessary to analyze the change in residual pesticide trends in leafy vegetables by comparing the recent analysis (after 2010) with the results from 10 years ago [13,20,21]. 

We conducted an analysis of 230 kinds of pesticide residues in 17,977 leafy vegetables (49 kinds) that were imported into Gwangju Metropolitan City (one of the largest consumption cities for leafy vegetables produced in Gwangju Metropolitan City and Chonnam Province) in South Korea over a period of 15 years (from 2005 to 2019). This study is expected to contribute to the management of residual pesticide leafy vegetables not only in South Korea but also on a global scale by analyzing the long-term trends and characteristics of the detection of residual pesticides.

## 2. Materials and Methods

### 2.1. Monitoring Programs and Sampling

A total of 17,977 samples were collected over 15 years (2005–2019). Sampling was conducted in keeping with the Korea Food Code guideline. The scope of our pesticide residue monitoring program was to analyze 49 types of leafy vegetables according to Table 1. The sampling plan consisted of two main parts. The first part involved sampling the incoming wholesale market before the produce was distributed in Gwangju Metropolitan City. The samples analyzed under the government surveillance program (The Health and Environment Research Institute under the Gwangju Metropolitan Government) were collected mainly from the two largest Gwangju area wholesale markets in Gwangju Metropolitan City and Chonnam Province from 2005 to 2019. The second part involved the sampling of leafy vegetables distributed in large or small local markets in Gwangju Metropolitan City. Most of the leafy vegetables consumed and distributed in South Korea were selected, and samples were collected. Whole samples were successfully analyzed within 24 h after collection.

### 2.2. Reagents and Sample Preparation

The Health and Environment Research Institute (Department of Pharmacochemistry) in Gwangju Metropolitan City conducted the tests up until 2010, and from 2011, the tests were conducted by the Agro-Fishery Products Inspection Center at the Health and Environment Research Institute in Gwangju Metropolitan City. This study used 230 certified pesticide reference standards purchased from Waco (Osaka, Japan) or Dr. Ehrenstorfer GmbH (Augsburg, Germany) (Table 2). Sample preparation was carried out with multiple reaction monitoring (MRM) No. 2 for pesticide residues in keeping with the Korea Food Code. Compounds and solvents (anhydrous sodium, dichloromethane, acetone, n-hexane, and acetonitrile) were obtained from Merck (Darmstadt, Germany). 

Samples (50 g) ground among the representative portion of the samples (sample size of each vegetable range: 1–2 kg) in a blender (Robot-coupe, South Perkins, Ridgeland, USA) were extracted with 100 ml acetonitrile for 2–3 min. The homogenized mixtures were filtered into a bottle with 10 g anhydrous sodium chloride. The extracts were vigorously vortexed for 1 min. Aliquots of 10 mL were transferred into a tube and evaporated to dryness with a gentle stream of air. Sample extracts for GC (gas chromatographic) analysis were dissolved in 4 ml of 20% acetone/hexane and loaded onto Florisil cartridges (Phenomenex, Torrance, CA, USA) (after activating and conditioning). The Florisil cartridges were eluted with 5 ml of 20% acetone/hexane again. The elutions were evaporated with a gentle stream of air and dissolved with 2 ml of acetone for GC analysis. For LC, sample extracts were dissolved with 4 ml of 1% methanol/dichloromethane and loaded onto SPE NH_2_ Cartridges (Phenomenex, Torrance, CA, USA) (after activating and conditioning). The SPE NH_2_ Cartridges were eluted with 7 ml of 1% methanol/dichloromethane again. The elutions were evaporated with a gentle stream of air and dissolved with 2 ml of methanol for LC analysis.

### 2.3. Instrumental Analysis

Instrumental analysis was performed using GC (gas chromatography) instruments and apparatuses and LC (liquid chromatography) instruments and apparatuses. The details were as follows: the GC-nitrogen phosphorous detector (NPD) system (for organophosphorus and nitrogen-containing compounds) and the GC-^63^Ni electron capture detector (ECD) system (for organochlorine and pyrethroid compounds) were used for GC analysis. An Agilent 7890 series GC instrument coupled to the NPD and ECD was used for analysis. The chromatographic separation was accomplished using a DB-5 column (Agilent, Santa Clara, CA, USA). The confirmation of residues was carried out with an Agilent 6890 series GC combined with an HP 5973 MSD (mass selective detector). A DB-5 (0.25 mm × 30 m, 0.25 μm film thickness; Agilent) column for GC-NPD and GC-ECD, and a DB-5MS (0.25 mm × 30 m, 0.25 μm film thickness; Agilent) column for GC-MS (mass spectrometry) were used for separation. The GC analysis conditions were as follows: 

1. GC-NPD: The column temperature was programmed from 190 to 240 °C (held for 3 min) with an increase of 4°C/min and increased to 290°C at 20°C/min (held for 5 min).

2. GC-ECD: The column temperature was programmed from 190 to 220 °C (held for 10 min) with an increase of 12°C/min and increased to 290°C at 7°C/min (held for 6 min).

3. GC-MS: The column temperature was programmed from 190 to 290 °C (held for 5 min) with an increase of 10°C/min.

LC analysis was performed using a Waters UPLC (Ultra Performance Liquid Chromatography) Acquity H-class (Waters Corporation, Milford, MA, USA) and a TSQ Quantum Ultra triple quadrupole mass spectrometer (Thermo Fisher Scientific, Miami, FL, USA). A reversed-phase Acquity UPLC BEH C18 LC column (2.1 × 50 mm, 1.7 μm; Acquity Group, Chicago, IL, USA) was used for chromatographic separation in UPLC. The TSQ Quantum Ultra triple quadrupole mass spectrometer and the UPLC were used in combination. Electrospray ionization was performed in positive and negative modes. Data were obtained in MRM mode. The capillary temperature of the MS was 300 °C, and the ion spray voltage was 3500 eV. Collision-induced dissociation was conducted with argon. Collision cell argon gas pressure was set to 1.5 mTorr. Chromatographic analyses were performed with gradient elutions (eluent A: formic acid (0.1%) and methanol–water (98:2, *v/v*), and eluent B: formic acid (0.1%) and methanol). Gradient elutions started at 95% of eluent A and 5% of eluent B for 0.2 min. This step was held for an additional 3.0 min before being returned to 100% of eluent B. The overall running time was 6 min. The separation of analytes was performed with a flow rate of 0.45 mL/min. The column temperature was maintained at 40 °C. The injection volume was 1 μL. 

### 2.4. Method Validation

Analysis of the lettuce matrix was used for validation of the sample preparation and analytical methods, linearity, LOD, LOQ, and recovery. The analytical methods validation was conducted according to the SANTE guidelines [22]. Since our study was a long-term study for 15 years, validation tests were carried out three times during the research period. This was conducted simultaneously with other previous studies conducted in our institution [13,20,21]. We used Excel 2016 (Microsoft, Redmond, WA, USA) to calculate the average value of residual pesticide detection and nonconformity.

## 3. Results and Discussion

### 3.1. Method Validation

A total of 19 different pesticides detected at more than 1% (pesticides <MRLs and >MRLs) were chosen for the method validation.

The validation results met the validation parameters and criteria (sensitivity, linearity, recovery, precision, and accuracy) in the SANTE guidelines [22]. The sensitivity of the method was confirmed with the LOD and LOQ. An S/N of 3:1 and 10:1, respectively, was accepted. The matrix-matched calibrations were obtained for the linearity test by plotting the area of each target pesticide against the concentrations of the calibration standards. Mean recoveries from the initial validation should be within the range 70–120%, with an associated repeatability RSD ≤ 20% for precision, for all analytes within the scope of a method, according to the SANTE guidelines [22]. The results shown in Table 3 met this criterion. Table 3 represents the results of the validation testing. The correlation coefficient showed good linearity of 0.9902‒0.9999. Percent recoveries were 86.2–109.5% for all pesticides. The standard deviation of the recovery rate was < 5.7%. The LOD of pesticides analyzed were from 0.003 to 0.043 μg g^–1^ and LOQ ranged between 0.010 and 0.129 μg g^–1^. Results from this study indicate that the analytical methods were appropriate for the analysis of pesticide residues in our study.

### 3.2. Trends in Annual Pesticide Residue Levels

**Figure 1:**
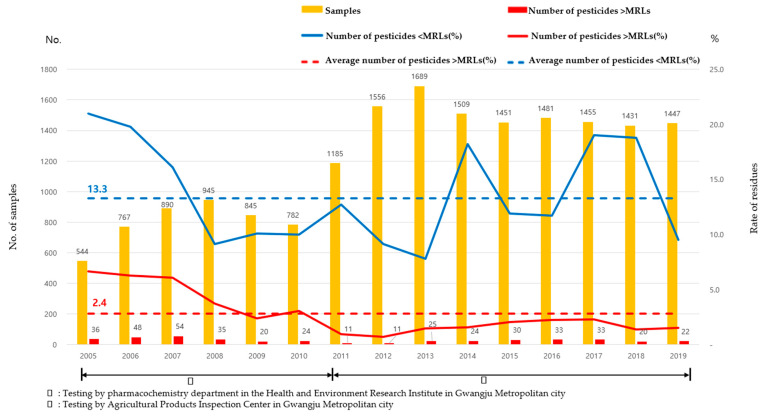
Samples analyzed by the monitoring programs of pesticide residues (Gwangju Metropolitan City) from 2005 to 2019: Trends in the annual number and percent (pesticides residues < MRLs and > MRLs).

This is the first long-term study of pesticide residues in leafy vegetables conducted in areas with the highest production volumes in South Korea. A total of 17.977 samples were analyzed for pesticide residues from 2005 to 2019 (Figure 1). A total of 4.773 samples were analyzed by the Health and Environment Research Institute (Department of Pharmacochemistry) (2005–2010), and 13.204 samples were analyzed by the Agro-Fishery Products Inspection Center established by the Health and Environment Research Institute Figure 1. (2011–2019).

Prior to the establishment of the inspection center, less than 1000 tests were conducted annually, between 544 and 945. In 2011, an agricultural inspection agency was established, and since then, the number of annual inspections has doubled. 

The number of samples with pesticide residues below the MRLs is 2.389 (13.3%) among the 17,977 leafy vegetable samples over the 15-year period. In addition, samples exceeding the MRLs total 426 (2.4%). From 2005 to 2007, residues below the MRLs tended to be higher than the average (Figure 1.). We can see that the nonconformity rate has decreased since 2005, and in particular, it has been below average since 2011. The number of samples with pesticide residues above MRLs was also higher than the average (2.4%) from 2005 to 2007. Overall, there was a high percentage of samples with pesticide residues below the MRLs and above the MRLs, between 2005 and 2007. It can be seen that the pattern of sample proportions with pesticide residues below the MRLs and above the MRLs is not consistent. Sample portions with pesticide residues below the MRLs were detected at a higher rate in 2014–2018 than in 2008–2013. However, the proportion of nonconformities remained below average between 2014 and 2018 (Figure 1). We think farmers used pesticides correctly according to the method of use. The rate of nonconformity was the highest in 2005, but it is decreasing, and it seems that it has been managed below the nonconformity average for the last nine years. The causes of the management below the nonconformity average are thought to be as follows. 

When nonconformities are detected, the following procedures are carried out in South Korea: The Agricultural Products Inspection Center conducts residual pesticide tests on agricultural products entering the market and agricultural products distributed in the region. When nonconformities are found in the tests, administrative actions are taken through the relevant county offices and district offices. Restrictions are imposed on farms that violate the law (treatment fees and suspension of shipments). The National Agricultural Products Quality Management Service in South Korea visits the farm and inspects the produce again. These efforts are combined to enable farmers to use pesticides accurately in South Korea. 

### 3.3. Evaluation of Pesticide Residue Levels by Pesticide Type

**Figure 2:**
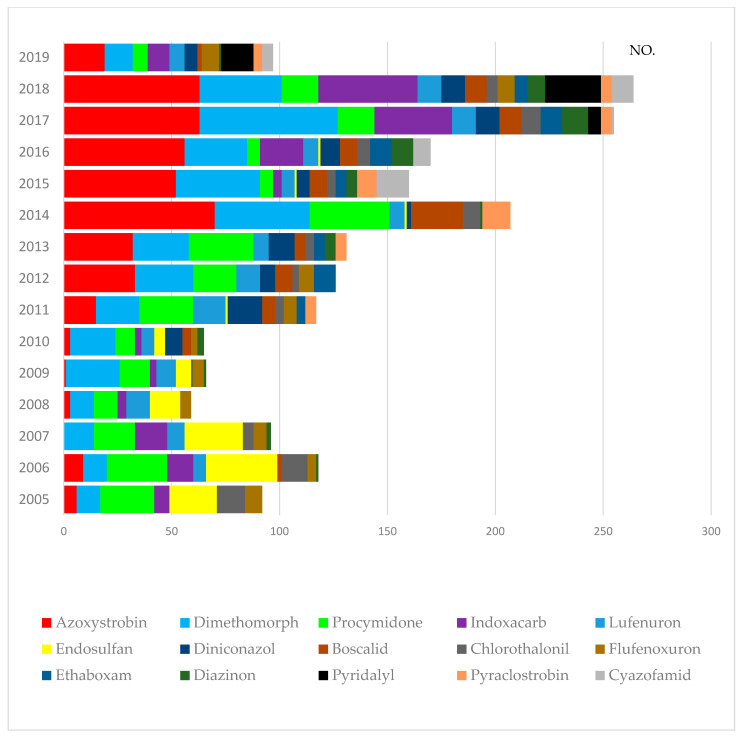
Distribution of pesticide residues (<MRLs) from 2005 to 2019: the 15 pesticides most frequently detected in the samples analyzed.

In 2.389 samples, we detected pesticide levels below the MRLs. Table 4 summarizes the main pesticides detected in leafy vegetables. Azoxystrobin (17.8%, 425/2.389), dimethomorph (16.5%, 393/2.389), procymidone (11.3%, 271/2.389), indoxacarb (6.7%, 160/2.389), and lufenuron (5.1%, 122/2.389) were the most frequently detected pesticides (with low MRLs). These types of pesticides account for nearly 58% of the total amount. However, Figure 2 shows that these values changed over the long term. The most frequently detected azoxystrobin was rarely detected before 2010. The detection increased after 2011, and it was frequently detected until 2018. Azoxystrobin is a broad-spectrum systemic fungicide of the class of methoxyacrylates (ARS 2008) [23]. Some pesticides, such as procymidone and endosulfan, were frequently detected, but our results show that their concentrations have decreased in recent years. Pesticides such as procymidone, widely used in South Korea, are managed through farm management by concerned authorities (The National Agricultural Products Quality Management Service, county offices, and district offices). Therefore, it seems that the frequency of detection of procymidone has decreased compared with that in the past. Endosulfan is a harmful agrochemical as an organochlorine insecticide and acaricide due to its acute toxicity and potential for bioaccumulation. Further, it is considered as a potential endocrine disruptor [24]. A worldwide ban on the production and use of endosulfan was agreed under the Stockholm Convention in April 2011 [25]. Since 2011, endosulfan has been banned from being manufactured and used in South Korea. The management system has been strengthened since 2015, as it was included in the Korean persistent organic pollutants (POPs) [26]. As shown in Figure 2, the use of endosulfan decreased after 2011. Despite the ban on the use of endosulfan, one detection per year in 2011 and 2014–2016 was made based on our research. Based on these results, these pesticides need to be managed continuously via official monitoring programs in case they are used. Indoxacarb was detected before 2014, from 2005 to 2010. It was not detected from 2011 to 2014 and has been detected again since 2015 (4–36 times per year). Pyridalyl was detected for the first time in our tests in 2017, and its detection (6–26 times per year) has increased in the last three years (2017–2019). 

The order of the high detection of pesticide residues (number of pesticides <MRLs) and the order of the high violations of the MRLs do not match in our study. The pesticides above the MRLs are procymidone (9.4%, 40/426), diniconazole (8.7%, 37/426), lufenuron (7.8%, 33/426), diazinon (7.5%, 32/426), and chlorpyrifos (7.0%, 30/426) (Table 4). 

The results of other studies of other countries around the world show that the most common pesticides detected in tested leafy vegetables were cyhalothrin and cypermethrin, in China [27]. In addition, chlorpyrifos and boscalid in Chile and chlorpyrifos in Thailand were detected mainly in leafy vegetables [28,29]. Pesticide residues detected in Denmark were different from our results. In the lettuce and spinach studied in Denmark, dithiocarbamates, cyprodinil and bromide ions, lambda-cyhalothrin, deltamethrin, and dithiocarbamates were found [30]. These results show that the pesticide components, which are mainly used in leafy vegetables, vary from country to country.

### 3.4. Violations of MRLs and Health Risk Assessment

In this study, 49 types of leafy vegetables (17,977 samples) were analyzed mainly for 230 pesticides. The number of pesticides over the MRLs in main leafy vegetables is described in Table 5. 

**Figure 3:**
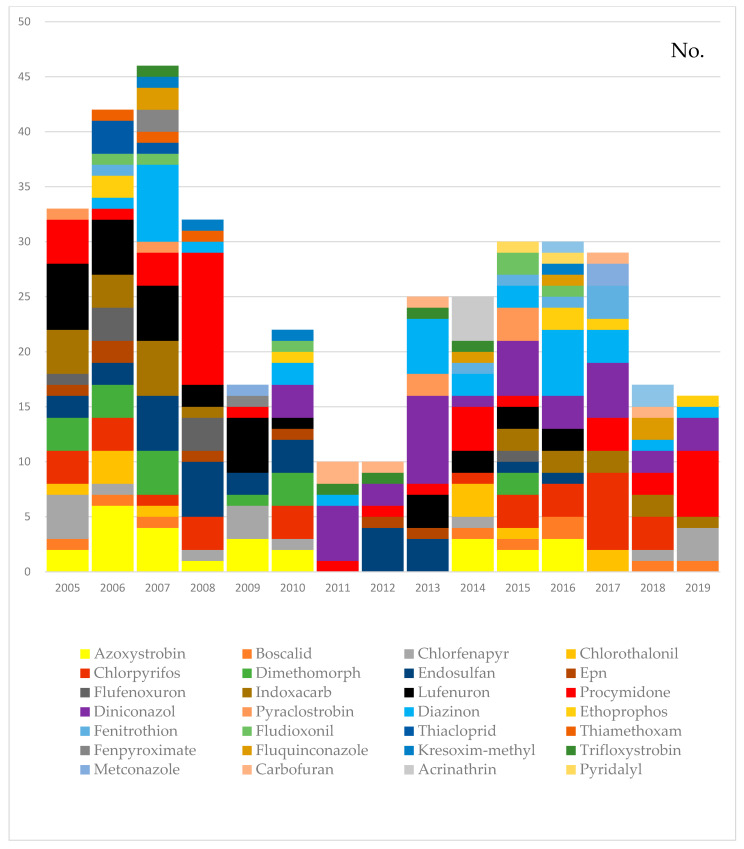
Annual distribution of pesticide residues exceeding the maximum residue limits (MRLs) in samples analyzed from 2005 to 2019.

A total of 61 pesticides (436 times) were detected in 426 samples that exceeded the MRLs. According to the food safety management guidelines, the top items above the MRLs in leafy vegetables in research from all over South Korea were as follows: perilla leaves, spinach, lettuce, napa cabbage, crown daisy, pepper leaves, pimpinella brachycarpa, chard [31,32,33]. These findings are generally consistent with the results of our study. However, there was one characteristic difference. In our results, ssamchoo (brassica lee ssp. namai) is the second highest in the leafy vegetables exceeding the MRLs. It is thought to be the reason why there were more inflows compared to other cities as a consumption site near the production area of ssamchoo (brassica lee ssp. namai). In studies of other countries around the world, the pesticides most frequently found above the MRLs in cabbage were mainly procymidone, in Brazilian monitoring programs [1]. Metalaxyl, boscalid, chlorothalonil, and difenoconazole exceeded the MRLs for chard, and mancozeb, difenoconazole, lambda-cyhalothrin, and thiamethoxam exceeded the MRLs for lettuce in Chile [28]. Additionally, the pesticides most frequently found above the MRLs in pak choi were chlorpyrifos in China [27]. We could see that procymidone, boscalid, chlorothalonil, and chlorpyrifos were the main pesticides, similar to our results, that caused nonconformities in leafy vegetables in other countries. Compared with Figure 3, the overall results of the country are similar to those of our study results between 2007 and 2012, with the highest detection of endosulfan as a whole, and procymidone, chlorpyrifos, diazinon, azoxystrobin, and diniconazole are also high. 

**Figure 4:**
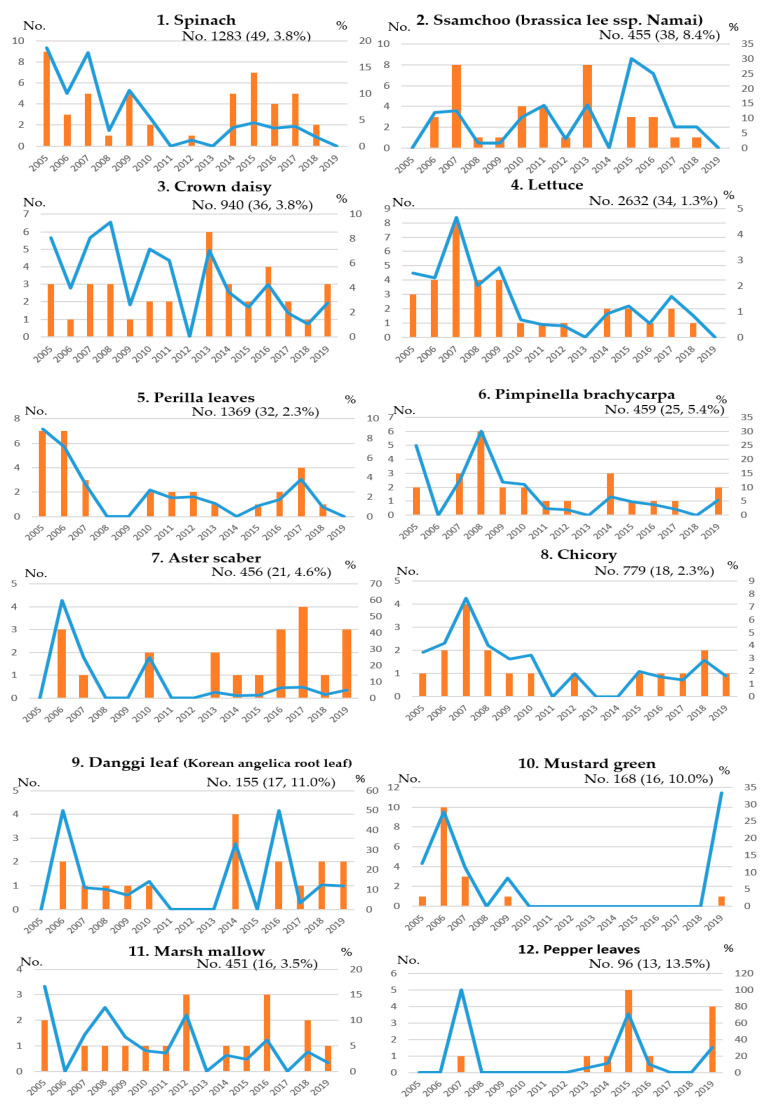
Annual distribution by which the MRLs are exceeded in the main leafy vegetables analyzed from 2005 to 2019 (blue lines—the nonconformity rate of samples, orange bars—the number of nonconformities of samples).

However, lufenuron was the highest detected pesticide in 2005 and 2009, with minor differences (Figure 3). In the long term, we can see that the trend of pesticides violating the MRLs changes, as shown in Figure 3. From 2005 to 2009, lufenuron, azoxystrobin, and diazinon were the top pesticides that violated the MRLs. Characteristically, from 2010, the proportion of lufenuron in nonconformities decreased. Further, diniconazole tended to account for a high proportion. As a result, there has been a change in the trend of pesticides violating the MRLs. Table 5 shows that the MRLs rate of certain agricultural chemicals is high for the following agricultural produce: chlorpyrifos, lufenuron in spinach, diniconazole in ssamchoo (brassica lee ssp. namai), procymidone in pimpinella brachycarpa, and aster scaber. These results indicate that certain leafy vegetables have been identified with violations of the MRLs for specific pesticides. Based on these results, leafy vegetables that were frequently detected with certain pesticides need to be intensively managed. Even with the same pesticide, various MRLs are set up for each agricultural product, and it seems to have been used without accurate information on agricultural products with exceptionally low MRLs. Ssamchoo (brassica lee ssp. namai) was developed in South Korea in 1998 and created through the interspecies hybridization of cabbages and napa cabbages. It is a new vegetable that has completely different ecological characteristics, shapes, and genetic composition than those of cabbage and Korean cabbage varieties [34]. In South Korean food culture, people wrap meat and rice in leafy vegetables. Therefore, if ssamchoo (brassica lee ssp. namai) grows too large, it is less commercialized. It is thought to use many pesticides, such as diniconazole, to control growth to a moderate size. Diniconazole is a broad-spectrum triazole fungicide that acts as a plant growth regulator, decreasing the height and leaf area in bean plants when applied to roots [35,36]. Figure 4 shows the trend of pesticides over the MRLs for each agricultural product. This shows the level of pesticide residue management for each vegetable in the long run.

For example, lettuce, spinach, and perilla leaves, the representative leafy vegetables consumed in South Korea, seem to be systematically managed because of the low number of violations of MRLs compared with that in the past. On the other hand, MRLs violations occur sporadically in crown daisy, aster scaber, danggi leaf (Korean angelica root leaf), marshmallow, and pepper leaves. Therefore, it is necessary to continuously check various vegetables for residual pesticides in order to prevent safety blind spots on agricultural products.

Based on our results, we conducted the exposure evaluation with pesticides that exceeded the MRLs. We chose four kinds of leafy vegetables (spinach, ssamchoo (brassica lee ssp. Namai), lettuce, perilla leaves). These vegetables with the most violations of MRLs in our study are consumed a lot in South Korea. A risk assessment was conducted with the estimated daily intake (EDI) and acceptable daily intake (ADI). The ADI is an estimate of the maximum amount of a substance consumed daily over a lifetime without an appreciable health risk [37]. 

The calculation of the EDI was made using the pesticide residue results and average food consumption per person per day [31]. The calculated results are demonstrated in Table 6.

The range of EDIs calculated was 1.0 × 10^–5^−8.4 × 10^–2^. The range of the hazard index was 0.001–7.6%. The ratio for chlorothalonil in spinach was 7.6% and the highest in the risk assessment test. A hazard index (percent of EDI to ADI ratios) over 100% indicates the possibility that the exposure would induce obvious toxic effects [38]. Further, leafy vegetables with the most violations of MRLs in our study in South Korea were not harmful to health by a risk assessment. However, as chronic exposure to pesticides may reduce cognitive abilities, the elderly and the weak should be careful about the intake and exposure of pesticides [39].

## 4. Conclusions

Among 17,977 samples, pesticides were detected in 2.815 samples (15.7%), of which 426 samples (2.4%) exceeded the MRLs. In our study, lettuce and perilla leaves, the representative leafy vegetables consumed in South Korea, seem to be managed due to the low number of violations of MRLs compared with that in the past. In our study, certain leafy vegetables were identified with the violation of MRLs for specific pesticides, and it seems that special care is needed. Moreover, leafy vegetables with the most violations of MRLs in our study in South Korea were not harmful to health by a risk assessment (the range of the hazard index was 0.001–7.6%). There were no results such as a long-term residual pesticide detection change as in our paper for leafy vegetables worldwide. Therefore, we think our results are a good prior study for the long-term study of residual pesticides in other countries. Further, because some leafy vegetables (spinach, napa cabbage, lettuce, etc.) in South Korea are exported abroad, our results are expected to be used as good data for importing countries. 

## Figures and Tables

**Table 1:** The scope of 49 types of leafy vegetables analyzed in our study.

Leafy Vegetable		
1. Winter-grown cabbage 2. Lettuce 3. Ssamchoo (brassica lee ssp. namai) 4. Bomdong (Korean spring cabbage) 5. Spinach 6. Perilla leaves 7. Crown daisy 8. Marshmallow 9. Young radish 10. Aster scaber 11. Pepper leaves 12. Danggi leaf (Korean angelica root leaf) 13. Pimpinella brachycarpa 14. Bok choy 15. Canola (leaf)	16. Mustard leaf 17. Kale 18. Shepherd’s purse 19. Chicory 20. Ssam cabbage 21. Cabbage 22. Mugwort 23. Chard 24. Butterbur 25. Narrow-head ragwort 26. Chickweed 27. Fischers ragwort 28. Red-veined sorrel 29. Sesame leaves 30. Toscana 31. Wild chive 32. Sedum 33. Arugula	34. Buckwheat leaves 35. Dandelion 36. Siler divaricata 37. Broccoli 38. Amaranth 39. Brassica campestris narinosa 40. Sugar beet leaves 41. Angelica 42. Napa cabbage 43. Ponytail radish 44. Oak leaf 45. Red oak lettuce 46. Parsley 47. Pumpkin leaves 48. Cabbage lettuce 49. Green mustard leaf

**Table 2:** Pesticides examined in our study (classified by pesticide class).

No.	Pesticide Class	Pesticides
1	1,3,5-triazine	terbuthylazine
2	2,6-dinitroaniline fungicide	fluazinam
3	Alkanamide (acetamide)	diphenamid
4	Anilide herbicides; phenoxy herbicides	clomeprop
5	Anilinopyrimidine	cyprodinil, mepanipyrim, pyrimethanil
6	Aromatic hydrocarbon; chlorophenyl/nitroaniline	dicloran, quintozene, tolclofos-methyl
7	Arylpyrrole	chlorfenapyr
8	Benzenesulfonamide	flusulfamide
9	Benzamide fungicide	zoxamide
10	Benzilate	bromopropylate
11	Benzoylurea	triflumuron, flufenoxuron, hexaflumuron, lufenuron, novaluron, teflubenzuron
12	Benzoisothiazole	probenazole
13	Carbamate	fenobucarb, furathiocarb, pirimicarb, aldicarb, bendiocarb, carbaryl, carbofuran, ethiofencarb, isoprocarb, methiocarb, methomyl, oxamyl, propoxur
14	Carboxamide	flutolanil, thifluzamide, mepronil, boscalid
15	Chloroacetamide	propisochlor, dimethenamid, thenylchlor
16	Chloronitrile	chlorothalonil
17	Cinnamic acid amide	dimethomorph
18	Cyclodiene organochlorine	BHC, endosulfan, chlordane
19	Diacylhydrazine	chromafenozide, methoxyfenozide, tebufenozide
20	Dicarboximide	iprodione, procymidone, vinclozolin
21	Dinitroaniline	pendimethalin
22	Dithiolane	isoprothiolane
23	Fungicides (aryl phenyl ketone fungicides)	metrafenone
24	Fungicides(sulfonamide fungicides; triazole fungicides)	amisulbrom
25	Hydroxyanilide	fenhexamid
26	Imidazole	imazalil, prochloraz, triflumizole
27	Isobenzofuranone	fthalide
28	Juvenile hormone mimic	fenoxycarb, pyriproxyfen
29	Methoxyacrylate	azoxystrobin
30	Methoxycarbamate	pyraclostrobin
31	Mite growth inhibitor	etoxazole
32	Neonicotinoid	acetamiprid, clothianidin, thiacloprid, thiamethoxam
33	N-phenyl carbamate fungicide	diethofencarb
34	N-phenylphthalimide	flumioxazin
35	Organophosphate	azinphos-methyl, EPN, ethion, mecarbam, methidathion, parathion-methyl, phenthoate, phosmet, phosphamidone, cadusafos, chlorpyrifos, chlorpyrifos-methyl, diazinon, dichlorvos, dimethoate, dimethylvinphos, ethoprophos, fenitrothion, fenthion, fosthiazate, malathion, parathion, phosalone, pirimiphos-methyl, profenofos, prothiofos, pyraclofos, quinalphos, tebupirimfos, terbufos, thiometon, triazophos
36	Phenylamide: butyrolactone	ofurace
37	Phenylamide: oxazolidinone	oxadixyl
38	Phenylpyrazole	fipronil
39	Oxadiazine	indoxacarb
40	Oxazolidinedione	pentoxazone
41	Oxime carbamate	butocarboxim, thiodicarb
42	Oximinoacetate	trifloxystrobin
43	Oxyacetamide	mefenacet, flufenacet
44	Phenylpyrrole	fludioxonil
45	Phthalimide	captafol, captan, folpet
46	Phosphorothiolate	edifenphos, iprobenfos, pyrazophos
47	Propionamide	fenoxanil
48	Pyrethroid	acrinathrin, bifenthrin, cyfluthrin, cyhalothrin, cypermethrin, deltamethrin, fenpropathrin, fenvalerate, permethrin, tefluthrin, tralomethrin
49	Pyridine	dithiopyr, thiazopyr
50	Pyrimidine	fenarimol, nuarimol
51	Pyrimidinol	bupirimate
52	Pyrimidinyloxybenzoic	pyriminobac-methyl
53	Pyrroloquinolinone	pyroquilon
54	Pyrazole herbicide	pyrazolate
55	Qil	cyazofamid
56	Strobilurin type- methoxyacrylate	picoxystrobin
57	Strobilurin type: oximinoacetate	kresoxim-methyl
58	Sulfamide	dichlofluanid
59	Sulfonylurea	cinosulfuron
60	Tetronic acid	spirodiclofen
61	Thiocarbamate	Esprocarb, molinate, pyributicarb
62	Thiazole carboxamide	ethaboxam
63	Thiazdiazole carboxamide	tiadinil
64	Triazolobenzothiazole	tricyclazole
65	Triazole	diniconazole, fenbuconazole, penconazole, triadimefon, uniconazole, cyproconazole, flusilazole, metconazole, myclobutanil, simeconazole, fluquinconazole, imibenconazole
66	Uracil	bromacil
67	Valinamide carbamate	iprovalicarb
68	Urea	methabenzthiazuron
69	The others	aldrin, binapacryl, butafenacil, carbophenothion, chinomethionat, chlorobenzilate, cyanazine, cyflufenamid, DDT, dicofol, dieldrin, diflufenican, dimethachlor, endrin, flonicamid, flumiclorac pentyl, fluthiacet-methyl, fluvalinate, heptachlor, indanofan, lactofen, mefenpyr-diethyl, methoxychlor, nitrapyrin, nitrothal-isopropyl, pyridalyl, pyrimidifen, tetradifon, ametryn, anilofos, azaconazole, cyanophos, dimepiperate, diphenylamine, etrimfos, fenazaquin, fenothiocarb, tolyfluanid, fonofos, isazofos, isofenphos, isoxathion, paclobutrazol, pirimiphos-ethyl, propazine, pyridaben, tebufenpyrad, triticonazole, 3,4,5-Trimethacarb, benzoximate, chlorantraniliprole, cymoxanil, fenpyroximate, ferimzone, fluacrypyrim, forchlorfenuron, metolcarb, oxaziclomefon, promecarb, pyribenzoxim, quinoclamine

**Table 3:** Validation parameters (linearity, LOD, LOQ, and recoveries) in main pesticides detected in our study.

Pesticide	Correlation Coefficient (r^2^)	LOD (μg g^–1^) ^a^	LOQ (μg g^–1^) ^b^	Recovery ± RSD (%)
Azoxystrobin	0.9991	0.041	0.124	90.1 ± 3.4
Boscalid	0.9998	0.020	0.059	94.1 ± 3.4
Chlorfenapyr	0.9978	0.038	0.033	105.5 ± 1.0
Chlorothalonil	0.9988	0.023	0.071	93.9 ± 1.7
Chlorpyrifos	0.9995	0.007	0.020	95.5 ± 3.2
Cyazofamid	0.9990	0.012	0.035	92 ± 2.1
Diazinon	0.9998	0.003	0.011	86.2 ± 3.8
Dimethomorph	0.9975	0.033	0.100	101 ± 1.9
Diniconazole	0.9992	0.008	0.026	99.5 ± 0.4
Endosulfan	0.9997	0.005	0.015	88.7 ± 0.6
Ethaboxam	0.9999	0.003	0.010	97 ± 1.2
Ethoprophos	0.9996	0.006	0.017	89.9 ± 3.9
Fenitrothion	0.9997	0.005	0.016	109.5 ± 2.7
Flufenoxuron	0.9992	0.043	0.129	100.0 ± 5.7
Indoxacarb	0.9994	0.007	0.021	102.0 ± 0.6
Lufenuron	0.9967	0.016	0.049	100.4 ± 2.7
Procymidone	0.9997	0.005	0.014	87.1 ± 1.6
Pyraclostrobin	0.9902	0.036	0.110	93 ± 5.5
Pyridalyl	0.9987	0.011	0.032	101.3 ± 4.1

^a^: limit of detection, ^b^: limit of quantification.

**Table 4:** Pesticides most frequently detected (number of pesticides <MRLs (%) and >MRLs (%)) in the samples analyzed in our study from 2005 to 2019.

Pesticide	Number of Pesticides < MRLs (%) (Total: 2.389)	Pesticide	Number of Pesticides > MRLs (%) (Total: 426)
Azoxystrobin	425 (17.8%)	Procymidone	40 (9.4%)
Dimethomorph	393 (16.5%)	Diniconazole	37 (8.7%)
Procymidone	271 (11.3%)	Lufenuron	33 (7.8%)
Indoxacarb	160 (6.7%)	Diazinon	32 (7.5%)
Lufenuron	122 (5.1%)	Chlorpyrifos	30 (7.0%)
Endosulfan	112 (4.7%)	Endosulfan	28 (6.6%)
Diniconazole	88 (3.7%)	Azoxystrobin	26 (6.1%)
Boscalid	87 (3.6%)	Indoxacarb	22 (5.2%)
Chlorothalonil	74 (3.1%)	Dimethomorph	16 (3.8%)
Flufenoxuron	60 (2.5%)	Chlorfenapyr	15 (3.5%)
Ethaboxam	50 (2.1%)	Chlorothalonil	11 (2.6%)
Diazinon	49 (2.1%)	Boscalid	9 (2.1%)
Pyridalyl	47 (2.0%)	Flufenoxuron	8 (1.9%)
Pyraclostrobin	46 (1.9%)	Ethoprophos	7 (1.6%)
Cyazofamid	39 (1.6%)	Fenitrothion	7 (1.6%)
The others	366 (15.3%)	The others	105 (24.6%)

**Table 5:** Main pesticides detected in the 15 highest leafy vegetables exceeded MRLs in our study from 2005 to 2019.

Vegetables	Samples Analyzed	Number of Pesticides (%) < MRL And > MRL	Number of Pesticides > MRL (%)	Main Pesticides Exceeded Mrls (No.)	Mrls (Mg Kg^–1^)	Range of Pesticides Found in Samples (Mg Kg^–1^)
Spinach	1.283	354 (27.6)	49 (3.8)	Chlorpyrifos (9)	0.01 → 0.05 *	0.06 ~ 0.92
				Lufenuron (9)	0.2 → 5.0 *	0.4 ~ 3.8
				Indoxacarb (6)	1.0 → 3.0 *	1.1 ~ 7.2
				Chlorothalonil(4)	5.0	5.5 ~ 22.1
Ssamchoo(brassica lee ssp. Namai)	455	119 (26.2)	38 (8.4)	Diniconazole (19)	0.3	0.4 ~ 6.6
				Diazinon (11)	0.1	0.2 ~ 3.4
Crown daisy	940	145 (15.4)	36 (3.8)	Diazinon (7)	0.1 → 0.01 *	0.09 ~ 1.79
				Lufenuron (4)	0.2 → 5.0 *	0.3 ~ 2.1
				Procymidone (3)	5.0 → 0.05 *	8.40 ~ 11.40
Lettuce	2.632	212 (8.1)	34 (1.3)	Endosulfan (4)	1.0 → 0.05 *	0.30 ~ 6.20
				Procymidone (4)	5.0	6.3 ~ 10.7
				Chlorpyrifos (3)	0.01	0.07 ~ 0.30
				Lufenuron (3)	0.2 → 7.0 *	0.4 ~ 2.1
Perilla leaves	1.369	324 (23.7)	32 (2.3)	Diniconazole (9)	0.3	0.7 ~ 2.4
				Azoxystrobin (4)	2.0 → 20 *	4.4 ~ 20.7
Pimpinella brachycarpa	459	132 (28.8)	25 (5.4)	Procymidone (15)	5.0 → 0.05 *	0.17 ~ 35.68
Aster scaber	456	157 (34.4)	21 (4.6)	Procymidone (5)	5.0 → 0.05 *	0.31 ~ 34.4
				Azoxystrobin (2)	3.0	4.0, 9.4
				Chlorpyrifos (2)	0.01	4.77, 0.34
				Diazinon (2)	0.1 → 0.01 *	1.3, 3.9
				Parathion (2)	0.3 → 0.01 *	0.60, 1.20
Chicory	779	71 (9.1)	18 (2.3)	Endosulfan (3)	0.1 → 0.05 *	0.20 ~ 2.00
				Procymidone (3)	5.0 → 0.05 *	3.22 ~ 9.50
Danggi leaf (Korean angelica root leaf)	155	41 (26.5)	17 (11.0)	Azoxystrobin (4)	2.0 → 20 *	3.0 ~ 12.3
				Chlorfenapyr (3)	0.5 → 0.7 *	1.3 ~ 1.8
				Procymidone (3)	5.0 → 0.05 *	0.41 ~ 12.50
Mustard green	168	47 (28.0)	16 (9.5)	Chlorpyrifos (3)	0.01 → 0.15 *	0.22 ~ 1.47
				EPN (2)	0.1 → 0.01 *	5.30, 6.50
				Lufenuron (2)	0.2 → 5.0 *	0.6, 0.8
Marsh mallow	451	62 (13.7)	16 (3.5)	Endosulfan (3)	1.0 → 0.05 *	0.30 ~ 1.8
				Trifloxystrobin (3)	0.5 → 20 *	1.1 ~ 1.5
Winter-grown cabbage	909	218 (24.0)	16 (1.8)	Endosulfan (3)	1.0 → 0.05 *	1.00 ~ 4.70
				Diazinon (2)	0.1	0.3, 0.7
				EPN (2)	0.2 → 0.01 *	4.1, 6.0
				Procymidone (2)	5.0 → 0.05 *	7.60, 15.80
Pepper leaves	96	37 (38.5)	13 (13.5)	Acrinathrin (3)	0.1 → 5.0 *	0.5 ~ 1.5
				Boscalid (3)	0.3 → 0.01 *	0.28 ~ 17.40
Young radish	678	164 (24.2)	13 (1.9)	Diazinon (3)	0.05 → 0.01 *	0.48 ~ 0.68
				Procymidone (2)	0.05	0.20, 0.51
				EPN (2)	0.05 → 0.01 *	7.10, 12.97
				Boscalid (2)	0.3	0.9, 3.0
Sesame leaves	312	70 (22.4)	10 (3.2)	Diazinon (2)	0.05 → 0.01 *	0.31, 3.14
				Diniconazole (2)	0.3	0.5, 0.6

Note: * Korean MRLs that have changed during our study period (2005–2019).

**Table 6:** Risk assessment of pesticides violating the MRLs in 5 main leafy vegetables.

Vegetables	Pesticide	Average Concentration (mg kg^–1^)	EDI (mg/ person/day) ^(a)^	ADI (mg/ person/day) ^(b)^	Hazard Index(%) ^(c)^
Spinach	Chlorpyrifos	0.32	0.00192	0.55	0.349091
	Lufenuron	1.47	0.00882	0.77	1.145455
	Indoxacarb	3.67	0.02202	0.55	4.003636
	Chlorothalonil	14.02	0.08412	1.1	7.647273
Ssamchoo (brassica lee ssp. Namai)	Diniconazole	1.38	0.00001	1.1	0.001255
	Diazinon	1.27	0.00001	0.275	0.004618
Crown daisy	Diazinon	1.27	0.00064	0.275	0.230909
	Lufenuron	1.45	0.00073	0.77	0.094156
	Procymidone	9.13	0.00457	5.5	0.083
Lettuce	Endosulfan	2.22	0.01288	0.33	3.901818
	Procymidone	8.3	0.04814	5.5	0.875273
	Chlorpyrifos	0.22	0.00128	0.55	0.232
	Lufenuron	1.06	0.00615	0.77	0.798442
Perilla leaves	Diniconazole	1.17	0.00304	1.1	0.276545
	Azoxystrobin	14.87	0.03866	11.0	0.351473

^(a)^ Average concentration (mg/kg) × Daily food intake (g/person/day)/1000. ^(b)^ Acceptable daily intake (mg/kgbw/day) × 55 kg. ^(c)^ Hazard index (%ADI) = (EDI/ADI) × 100.

## Data Availability

The datasets generated and/or analysed during the current study are not publicly available due data are not public but are available from the corresponding author on reasonable request.
